# Understanding the Role of the Antioxidant System and the Tetrapyrrole Cycle in Iron Deficiency Chlorosis

**DOI:** 10.3390/plants8090348

**Published:** 2019-09-13

**Authors:** Carla S. Santos, Rengin Ozgur, Baris Uzilday, Ismail Turkan, Mariana Roriz, António O.S.S. Rangel, Susana M.P. Carvalho, Marta W. Vasconcelos

**Affiliations:** 1 Universidade Católica Portuguesa, CBQF - Centro de Biotecnologia e Química Fina – Laboratório Associado, Escola Superior de Biotecnologia, Rua Diogo Botelho 1327, Porto 4169-005, Portugal; cssantos@porto.ucp.pt (C.S.S.); mroriz@porto.ucp.pt (M.R.);; 2 Department of Biology, Faculty of Science, Ege University, Bornova, İzmir 35100, Turkeyismail.turkan@ege.edu.tr (I.T.); 3 GreenUPorto – Research Centre for Sustainable Agrifood Production, Faculty of Sciences of University of Porto, Rua da Agrária 747, 4485-646 Vairão, Portugal

**Keywords:** ∂-aminolevulinic acid, FeSOD, heme-containing enzymes, hemin, oxidative stress

## Abstract

Iron deficiency chlorosis (IDC) is an abiotic stress often experienced by soybean, owing to the low solubility of iron in alkaline soils. Here, soybean lines with contrasting Fe efficiencies were analyzed to test the hypothesis that the Fe efficiency trait is linked to antioxidative stress signaling via proper management of tissue Fe accumulation and transport, which in turn influences the regulation of heme and non heme containing enzymes involved in Fe uptake and ROS scavenging. Inefficient plants displayed higher oxidative stress and lower ferric reductase activity, whereas root and leaf catalase activity were nine-fold and three-fold higher, respectively. Efficient plants do not activate their antioxidant system because there is no formation of ROS under iron deficiency; while inefficient plants are not able to deal with ROS produced under iron deficiency because ascorbate peroxidase and superoxide dismutase are not activated because of the lack of iron as a cofactor, and of heme as a constituent of those enzymes. Superoxide dismutase and peroxidase isoenzymatic regulation may play a determinant role: 10 superoxide dismutase isoenzymes were observed in both cultivars, but iron superoxide dismutase activity was only detected in efficient plants; 15 peroxidase isoenzymes were observed in the roots and trifoliate leaves of efficient and inefficient cultivars and peroxidase activity levels were only increased in roots of efficient plants.

## 1. Introduction

Iron (Fe) is an essential micronutrient required for the plant growth, being involved in several metabolic processes, including photosynthesis, respiration, nitrogen fixation, DNA synthesis, hormone production, and chlorophyll biosynthesis [1]. Although Fe is present in sufficient amount in the soil, under alkaline conditions its bioavailability is limited, resulting in the appearance of iron deficiency chlorosis (IDC).

**Figure 1:**
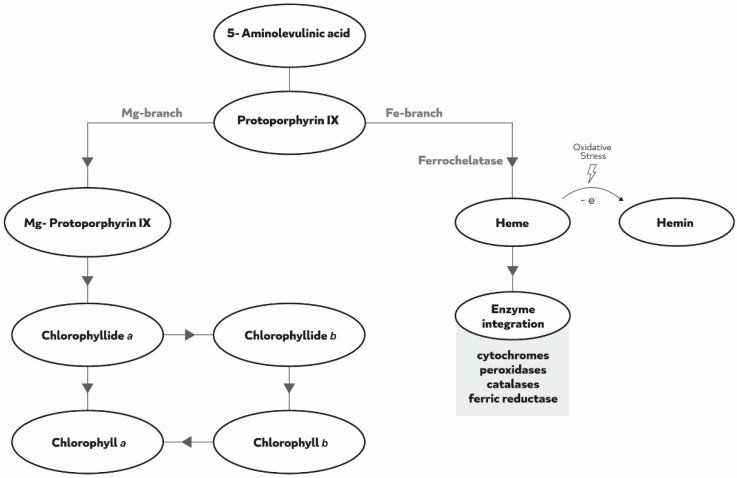
Schematic overview of the tetrapyrrole cycle.

Soybean (*Glycine max* L.) is the most important legume crop with an estimated world production of more than 350 million tons in 2017 [2]. Several crops are highly affected by IDC; however, one of the most susceptible crops, especially at early developmental stages, is soybean. IDC symptoms are characterized by yellowing of the upper leaves, interveinal chlorosis, and reduced growth and yield [3]. When there is a depletion of Fe, chlorophyll and other photosynthetic pigments, like anthocyanins and carotenoids, decreases as Fe is essential for their biosynthesis [3]. Because symptoms in soybean are so pronounced and because the economic consequences are so severe (yield losses have been estimated in excess of $120 million annually in the western Corn Belt and Great Plain Regions of the United States) [4], the vast majority of studies dealing with IDC have been published in soybean. The higher susceptibility of certain soybean lines to IDC is multifactorial and mainly because of genetic factors [5], but the physiological and biochemical underlining factors are still poorly understood. Soybean cultivars have been differentiated regarding their IDC susceptibility, where Fe-efficient plants activate biochemical reactions to make Fe more bioavailable, and Fe-inefficient do not [6], reinforcing the interest of this crop as a model system for studies regarding Fe-uptake efficiency [7]. The main biochemical reaction induced by dicotyledonous plants to cope with Fe deficiency is a reduction-based strategy for iron absorption (Strategy I) leading to the reduction of Fe^3+^ to Fe^2+^ by a root cell plasma membrane ferric reductase (like ferric reductase oxidase, FRO), and Fe^2+^ transport to the cytoplasm via iron regulated transporters [8,9], thus increasing Fe availability for the plant. One important characteristic of the FRO enzymes, in the context of Fe nutrition, is that they have a heme group as a constituent, which is essential for their functioning [9]. In turn, heme is produced via the tetrapyrrole cycle and Fe is essential for its biosynthesis [10]. Briefly, this cycle occurs mainly in the plastids, where 5-aminolevulinic acid (ALA) is synthesized via the conversion of glycine and succinyl-CoA by ALA synthase, and later on is converted to protoporphyrin IX [11,12] (Figure 1). The cycle is then divided in two branches, the “magnesium-branch” that leads to the synthesis of chlorophyll, and the “iron-branch” that leads to the formation of heme. In the first branch, Mg^2+^ is inserted into the backbone of proto forming Mg-protoporphyrin IX, which, after a series of modifications, forms chlorophyllide *a* that is esterified to synthesize chlorophyll *a*. Chlorophyllide *a* can also be converted into chlorophyllide *b*, forming chlorophyll *b* which can be converted again into chlorophyll *a*, forming the chlorophyll cycle. In the second branch, a ferrochelatase is responsible for inserting Fe^2+^ into proto to form heme *b* (protoheme) [11,13]. Once it is produced, heme is incorporated in several enzymes of the antioxidant system and Fe metabolism pathways [11] (Figure 1). It is known that heme suffers degradation when exposed to oxidative stress, being oxidized into its ferric form hemin [14], that is also pro-oxidant [15].

Iron is a constituent of the electron transport chain in mitochondria and chloroplasts, and its deficiency causes changes in cellular redox, resulting in reactive oxygen species (ROS) accumulation [16]. It has been shown that Fe-deficient plants are ROS producers [17,18,19,20], probably because Fe is a cofactor in ROS-detoxifying enzymes. Heme is also a necessary cofactor for the proper functioning of these enzymes [21], and lower levels of heme under Fe deficiency may further exacerbate ROS accumulation. To cope with oxidative stress and regulate ROS levels, plants have developed the antioxidant system comprising two levels of regulation, mediated by: (i) enzymes, namely, superoxide dismutase (SOD), catalase (CAT), peroxidase (POX), ascorbate peroxidase (APX), glutathione reductase (GR) and others, such as guaiacol peroxidase (GPX), monodehydroascorbate reductase (MDHAR) and dehydroascorbate reductase (DHAR) of which SOD, CAT and APX are heme containing proteins or use Fe as a cofactor; and (ii) metabolites, like ascorbate (ASC), glutathione (GSH), phenolics and carotenoids [22] among others [16,23,24,25]. SODs dismutate O_2_^−^ to H_2_O_2_, the major ROS produced in electron transport chains in both chloroplasts and mitochondria [16]. Moreover, SODs are classified into three classes according to the metal co-factor found in the active site of the enzyme, which are MnSOD, Cu/ZnSOD, and FeSOD. In particular, FeSOD is located in the chloroplasts and is an important regulator of the antioxidant response against abiotic factors [26]. CAT catalyzes the conversion of hydrogen peroxide (H_2_O_2_) to H_2_O [27], being an important part of the plant antioxidant system, and, like FRO, it is also a heme-dependent enzyme [28]. APX, also a heme containing enzyme, catalyzes the reduction of H_2_O_2_ to H_2_O, doing so through the oxidation of ASC and it is highly substrate specific, requiring reducing power for its functioning, being particularly associated with enhanced tolerance against abiotic stress [29]. Lower APX activities are associated with Fe-sensitive plants [30]. GR is involved in defense against oxidative stress and regenerates GSH from its oxidized form, allowing the ASC-GSH cycle to proceed [31]. Reports show that GR activity varies depending on the mineral stress to which the plants are subjected and it has been suggested that under Fe deficiency the activity of this enzyme may be increased [32,33].

Few studies have evaluated the relationship between the tolerance to Fe deficiency, the triggering of the tetrapyrrole cycle and the antioxidant defense mechanism in plants (recently reviewed in [21]). In the present study, we hypothesize that Fe efficiency, being linked to Fe accumulation and transport to the aerial organs, will be the key in the antioxidant system and tetrapyrroles synthesis regulation, through the activity of several heme (SOD, CAT, APX, and FRO) and non-heme (GR) containing enzymes. Here, inefficient plants, which are generally classified as unable to activate Fe-uptake mechanisms, are expected to suffer from greater oxidative damage, because of their inability to activate ROS scavenging enzymes, hence having lower antioxidative responses; while efficient plants, that activate the necessary biochemical reactions to make Fe available for absorption, should be able to properly activate the ROS scavenging enzymes, resulting in lower oxidative stress. We also hypothesize that efficient and inefficient plants differently regulate the SOD and POX isoenzymes, in order to better control the oxidative damage. To verify this hypothesis, the responses of two soybean lines with contrasting susceptibilities to Fe stress were evaluated by analyzing the morphological, physiological, and biochemical parameters. The constituents of the tetrapyrrole cycle were evaluated (ALA, total chlorophyll and heme in its oxidized form), as well as the photosynthetic pigments anthocyanins and carotenoids. In order to evaluate the oxidative stress of the plant tissues, lipid peroxidation was measured as the amount of thiobarbituric acid reactive substances (TBARS).

## 2. Results

### 2.1. Growth and Chlorosis Evaluation

**Figure 2:**
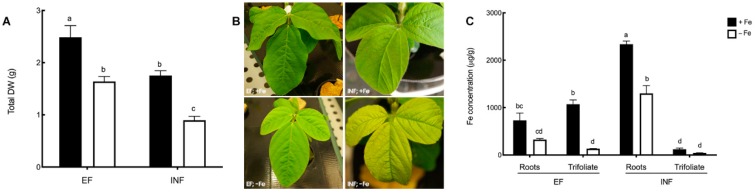
Morpho-physiological effects of Fe deficiency in efficient (EF) and inefficient (INF) soybean lines. (**A**) Total dry weight (DW); (**B**) chlorosis symptoms; (**C**) Fe concentration (µg/g) in roots and trifoliate leaves. Plants were grown under Fe sufficiency (+Fe, 20 µM) or Fe deficiency (−Fe, no Fe) for 14 days under hydroponic conditions. Data are means ± SE; different letters indicate significant differences (*p* < 0.05) by ANOVA with Tukey correction test.

Fe stress led to a decrease in the total dry weight in both efficient and inefficient plants. Inefficient plants under Fe sufficiency and Fe deficiency had the lowest total plant DW (1.8 ± 0.09 g and 0.90 ± 0.08 g, respectively), which corresponded to about half of the DW observed in efficient plants (Figure 2A). Visible interveinal chlorosis with remaining green veins was apparent in both lines under Fe deficiency, but was more acute in inefficient plants, confirming their initial classification (Figure 2B).

As expected, Fe concentration was about two-times lower in Fe-stressed roots of both efficient and inefficient plants, when compared to the Fe-sufficient plants (Figure 2C). In inefficient plants, Fe was mostly accumulated in the root tissues, with very low levels of leaf Fe concentration, independent of the Fe treatment. In contrast, efficient plants had higher concentrations of Fe in the leaves and no significant differences were found between this organ and the roots.

### 2.2. ALA and Photosynthetic Pigments Evaluation

**Figure 3:**
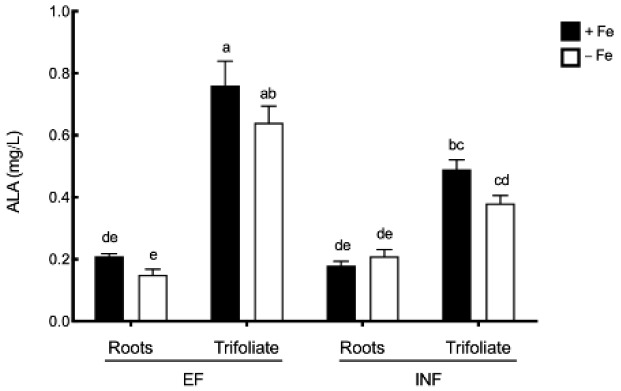
∂-aminolevulinic acid (ALA) concentration (mg/L) in roots or trifoliate leaves of efficient (EF) and inefficient (INF) soybean lines. Plants were grown under Fe sufficiency (+Fe, 20 µM) or Fe deficiency (−Fe, no Fe) for 14 days under hydroponic conditions. Data are means ± SE; different letters indicate significant differences (*p* < 0.05) by ANOVA with Tukey correction test.

Although Fe stress did not cause a significant effect on ALA concentrations, the inefficient plants accumulated 40% less ALA than the efficient plants in the trifoliate leaves (Figure 3). 

**Figure 4:**
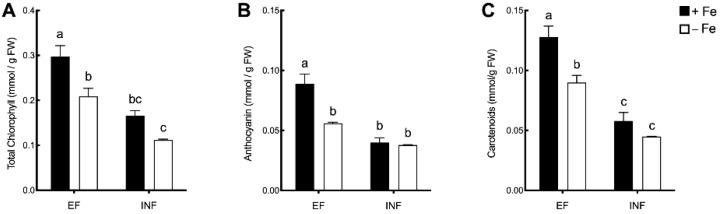
Photosynthetic pigment concentrations (mmol/g FW) in the trifoliate leaves of efficient (EF) and inefficient (INF) soybean lines grown under Fe sufficiency (+Fe, 20 µM) or Fe deficiency (−Fe, no Fe) for 14 days under hydroponic conditions. (**A**) Total chlorophyll; (**B**) anthocyanin; (**C**) carotenoids concentrations. Data are means ± SE; different letters indicate significant differences (*p* < 0.05) by ANOVA with Tukey correction test.

Total chlorophyll (Figure 4A), anthocyanin (Figure 4B), and carotenoid (Figure 4C) concentrations were evaluated. In the efficient plants, Fe stress induced decreases between 30% and 39% of these pigment concentrations, but in the inefficient plants, Fe availability did not significantly affect the photosynthetic pigments accumulation. On the other hand, inefficient plants presented significantly lower total chlorophyll (Figure 4A) and carotenoids (Figure 4C) concentrations when compared to the efficient plants.

### 2.3. Oxidative Stress Evaluation

**Figure 5:**
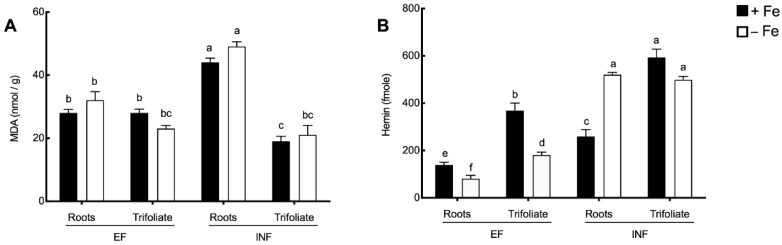
Malondialdehyde (MDA) concentration (nmol/g) and hemin concentration (fmole) in the roots and trifoliate leaves of efficient (EF) and inefficient (INF) soybean lines grown under Fe sufficiency (+Fe, 20 µM) or Fe deficiency (−Fe, no Fe) for 14 days under hydroponic conditions. (**A**) MDA; (**B**) hemin concentrations. Data are means ± SE; different letters indicate significant differences (*p* < 0.05) by ANOVA with Tukey correction test.

As an approach to the analysis of the oxidative stress in the tissues, lipid peroxidation and the concentration of the oxidized form of heme–hemin were evaluated (Figure 5A,B, respectively). Fe availability had no significant effect on MDA accumulation (Figure 5A). However, MDA values of inefficient plants were about 55% higher than those registered for efficient plants in the roots (*p* < 0.0001). In contrast, in the trifoliate the opposite trend was found, with a higher MDA concentration (20% increase) in the efficient line but only under Fe sufficiency. 

When looking at hemin concentration (Figure 5B), under Fe deficiency, it was significantly decreased in efficient roots and leaves, but significantly increased in double in inefficient roots. Additionally, hemin concentration was always higher in inefficient tissues when compared to the efficient counterparts (*p* < 0.05).

### 2.4. Enzymatic Activity

**Figure 6:**
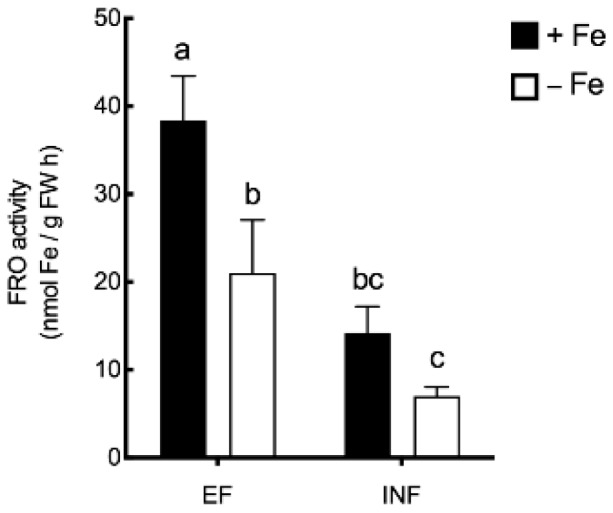
Root FRO activity (nmol Fe/g FW h) of efficient (EF) and inefficient (INF) soybean lines grown under Fe sufficiency (+Fe, 20 µM) or Fe deficiency (−Fe, no Fe) for 14 days under hydroponic conditions. Data are means ± SE; different letters indicate significant differences (*p* < 0.05) by ANOVA with Tukey correction test.

Iron stress caused a significant decrease in FRO activity of efficient plants but no significant changes were induced in the inefficient plants. Inefficient plants presented significantly lower levels of FRO activity when compared to the efficient plants (Figure 6). Under Fe stress, FRO activity of inefficient plants was of 0.007 ± 0.001 µmol Fe/g FW h, which was three-times lower than that of the efficient plants (0.021 ± 0.005 µmol Fe/g FW h).

**Figure 7:**
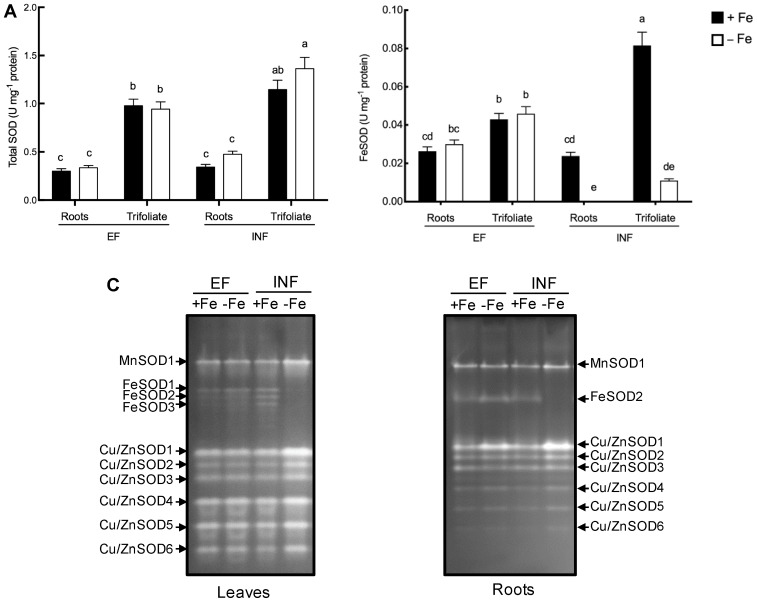
Total SOD activity and isoenzyme patterns of the roots and trifoliate leaves of efficient (EF) and inefficient (INF) soybean lines. Plants were grown under Fe sufficiency (+Fe, 20 µM) or Fe deficiency (−Fe, no Fe) for 14 days under hydroponic conditions. (**A**) Total SOD activity; (**B**) FeSOD activity; (**C**) leaves and roots SOD isoenzyme patterns. Data are means ± SE; different letters indicate significant differences (*p* < 0.05) by ANOVA with Tukey correction test.

To better understand the dynamics of O_2_^−^ scavenging upon Fe deficiency, SOD activity was analyzed (Figure 7) and the pattern of SOD isoenzymes was also investigated. Total root SOD activity was similar between efficient and inefficient plants, in both Fe treatments. However, under Fe deficiency, the inefficient cultivar had 31% higher SOD activity in trifoliate leaves compared to the efficient one (Figure 7A). Efficient plants were able to maintain FeSOD activity in both Fe treatments, while in inefficient plants FeSOD activity was null in the roots and decreased seven-fold in the leaves under Fe deficiency (Figure 7B). Concordantly, there were clear changes in SOD isoenzyme patterns (Figure 7C). A total of one MnSOD, three FeSOD, and six Cu/ZnSOD isoenzymes were observed. FeSOD isoenzymes were lost under Fe stress both in roots and trifoliate leaves of inefficient cultivar, while the same phenomenon was not observed in the efficient cultivar. On the other hand, other isoenzymes such as Cu/ZnSOD1 were induced by Fe stress, explaining the unchanged total SOD activity. 

**Figure 8:**
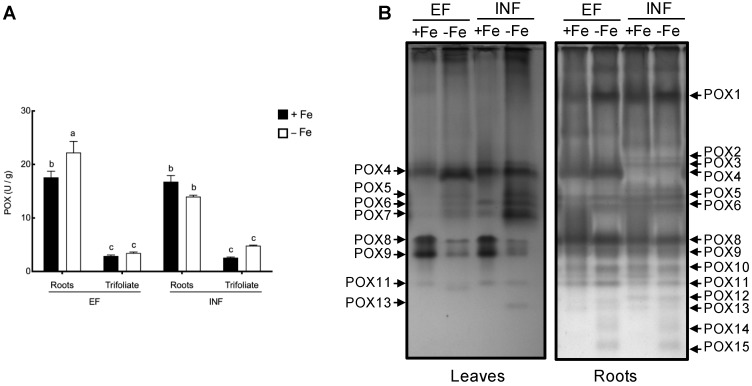
Total POX activity and isoenzyme patterns of the roots and trifoliate leaves of efficient (EF) and inefficient (INF) soybean lines. Plants were grown under Fe sufficiency (+Fe, 20 µM) or Fe deficiency (−Fe, no Fe) for 14 days under hydroponic conditions. Data are means ± SE; different letters indicate significant differences (*p* < 0.05) by ANOVA with Tukey correction test.

POX activity levels only increased in roots of efficient plants with Fe stress (by 26%), otherwise no significant effect of the Fe treatment was observed. Also, under Fe deficiency, the roots of efficient plants had 37% higher POX activity than the roots of the inefficient plants. Total of 15 different isoenzymes of POX were observed in the roots and trifoliate leaves of the efficient and inefficient cultivars (Figure 8). 

Under Fe deficiency total POX activity was significantly increased only in the efficient plant roots. In shoots, Fe deficiency did not induce any significant changes in the total POX activity (Figure 8A). However, cultivar, tissue, and Fe treatment-dependent changes in POX isoenzyme pattern were observed (Figure 8B). In roots, POX14 and POX15 were exclusively expressed under Fe stress in both cultivars, whereas POX2 and POX3 were only observed in inefficient plants; POX8 and POX9 had a drastic decrease of expression under Fe deficiency in the leaves of both lines.

**Figure 9:**
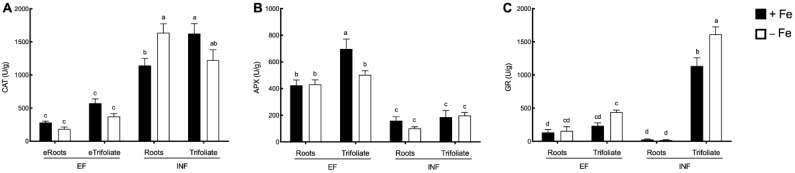
Enzyme activity in the roots and trifoliate leaves of efficient (EF) and inefficient (INF) soybean lines. (**A**) catalase activity (CAT); (**B**) ascorbate peroxidase activity (APX); (**C**) glutathione reductase activity (GR). Plants were grown under Fe sufficiency (+Fe, 20 µM) or Fe deficiency (−Fe, no Fe) for 14 days under hydroponic conditions. Data are means ± SE; different letters indicate significant differences (*p* < 0.05) by ANOVA with Tukey correction test.

Under Fe deficiency, roots of the inefficient plants increased CAT activity by 30% (Figure 9A). CAT levels were highly increased in the inefficient plants when compared to the efficient ones (Figure 9A). 

APX presented an opposite pattern to CAT, being significantly lower in the inefficient plants when compared to the efficient ones, with no significant changes registered between Fe treatments (Figure 9B).

Fe deficiency led to a 30% increase of GR activity in the trifoliate leaves of the inefficient plants (Figure 9C). Concerning the activity of this enzyme in the efficient plants no significant changes were registered between Fe treatments both in roots and shoots. Finally, in the trifoliate leaves, GR activity was significantly induced in the inefficient plants when compared to the efficient ones (Figure 9C). 

### 2.5. Principal Component Analysis

**Figure 10:**
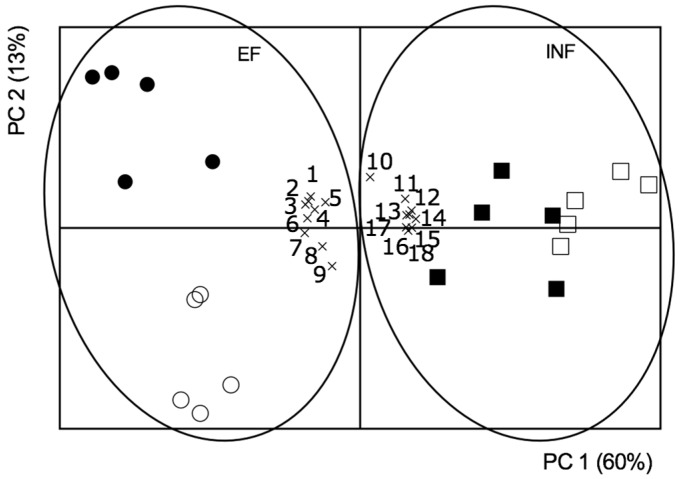
Biplot of score and loading factors of the principal component analysis (PCA). Efficient (circles) and inefficient (squares) soybean lines, grown under Fe sufficiency (+Fe, 20 µM; solid symbols) or Fe deficiency (−Fe, no Fe; open symbols) for 14 days under hydroponic conditions and associated factors: 1—anthocyanin concentration; 2—total chlorophyll concentration; 3—carotenoid concentration; 4—leaf ∂-aminolevulinic acid concentration; 5—leaf MDA concentration; 6—leaf ascorbate peroxidase activity; 7—root ascorbate peroxidase activity; 8—root glutathione reductase activity; 9—root reductase activity; 10—root ∂-aminolevulinic acid concentration; 11—leaf hemin concentration; 12—root hemin concentration; 13—leaf catalase activity; 14—root catalase activity; 15—leaf glutathione reductase activity; 16—root MDA concentration; 17—leaf SOD activity; 18—root SOD activity.

A PCA model was performed to extract the most important information from the current data set. The resulting components explained 73% of the variance (Figure 10). 

When analyzing the score plot of PC1 vs. PC2 (Figure 10) it was found that samples corresponding to efficient or inefficient plants were separated along the PC1 (60% of total variance) and samples of the efficient plants were further separated along the PC2 (13% of total variance), according to the Fe treatment.

Moreover, a high correlation between the photosynthetic pigments, leaf ALA concentration, APX activity (leaves and roots), leaf MDA concentration, GR activity in the roots, and FRO activity was observed. These factors were also highly correlated to the efficient plants. On the other hand, root ALA concentration, hemin concentration (leaves and roots), CAT activity (leaves and roots), leaf GR activity, root MDA concentration, and SOD activity (leaves and roots) were grouped, being correlated to the inefficient plants. 

Additionally, for the efficient plants, two correlation levels were found, depending on Fe stress: Under Fe sufficiency, there was a correlation with leaf photosynthetic pigments, MDA concentration, ALA concentration, and activity of APX; under Fe deficiency a correlation was found with root APX, GR, and FRO activities.

## 3. Discussion

In calcareous soils where high pH and bicarbonate ion concentrations are prevalent, Fe uptake is impaired given that, under such conditions, Fe is mostly in its ferric form (Fe^3+^) which is not bioavailable for direct plant absorption [3] causing severe yield losses in different crops worldwide. One of the possible strategies to reduce this problem is to select tolerant or efficient cultivars that are able to sustain Fe-deprivation stress [34]. As aforementioned, the definition for this Fe efficiency comprises the ability to induce biochemical reactions that make Fe available in a useful form [6]. However, this definition still lacks information on other factors that could contribute to this trait and recent studies have shown the importance of physiological [7,35] and molecular [36] mechanisms in the Fe efficiency trait of soybean plants. Meanwhile, other studies reported an induction of oxidative stress related reactions when Fe is unavailable for plant uptake and mobilization, since this nutrient is essential for a vast number of biological processes [37,38,39]. Plus, it has been shown that Fe uptake and tetrapyrrole biosynthesis are co-regulated [40].

The ability to induce the antioxidant machinery could have an important role in the Fe efficiency trait and tetrapyrroles were proposed as the signaling molecules for oxidative stress [21]. In this study, an integrative overview looking at tetrapyrrole cycle constituents (ALA, chlorophylls and heme in its oxidized form) as well as heme and Fe-cofactor containing enzymes was undertaken to understand the differences between two soybean lines with contrasting susceptibilities to Fe limitation. First, the difference in susceptibility to Fe stress was evaluated looking at the main symptoms associated to IDC, namely, stunted growth and interveinal chlorosis. Inefficient plants were smaller and displayed more noticeable visual IDC symptoms than the efficient plants, which confirmed previous studies using these accessions [36]. In the current study, the efficient line had higher levels of FRO activity in the roots when compared to the inefficient one, which suggests a higher Fe absorption. Once in the roots, Fe must be translocated to the shoots. Previous works have shown that inefficient soybean lines have less Fe translocation ability and tend to accumulate most of the absorbed Fe in the root tissue [36,37]. This was also true in the present study, showing that the ability to reduce and translocate Fe to the upper organs could be one of the major contributors for Fe-stress tolerance. The network of Fe transporters from the root to the shoot are well described [41], and may be linked to changes in organic acid metabolism, which are known to be correlated with Fe transport within the plant [42].

The response of the efficient and inefficient soybean cultivars to Fe deficiency, regarding ALA accumulation, was evaluated. Here, Fe deficiency did not have a significant effect on ALA accumulation, but the inefficient plants had lower leaf ALA concentrations when compared to the efficient plants. The reactions for ALA synthesis occur in the stroma of chloroplasts [43] and, since inefficient plants displayed more acute IDC symptoms and leaf damage, synthesis of ALA could be impaired, while efficient plants (that were greener and healthier) could allow for a more active synthesis of this product. Also, under Fe deficiency, inefficient plants have lower Fe concentration in their leaves which can decrease biosynthesis of ALA, since Fe stress downregulates the heme biosynthetic genes [12]. ALA is a precursor for chlorophyll biosynthesis, and a positive feedback correlation between these two metabolites has been reported [44,45,46]. Chlorophyll levels were significantly reduced in the shoots of the efficient cultivar because of Fe deficiency, whereas in the inefficient cultivar the reduction was not significant. This is in accordance to previous studies [36] and could be a mechanism used by the efficient plants for ROS control under stress, since decreasing chlorophyll lowers photoinhibition levels that would lead to ROS production [47]. The lower chlorophyll levels in the shoots of the inefficient line under Fe sufficiency may be a consequence of its inability to translocate Fe to the aerial parts, a necessary step for chlorophyll biosynthesis. Given the fact that Fe is essential for chlorophyll biosynthesis, other photosynthetic pigments are expected to decrease under Fe stress, such as anthocyanins and carotenoids [3]; however, in the inefficient plants this was not observed. The maintenance of anthocyanin and carotenoid concentrations under Fe limitation in inefficient plants could be a strategy for protection against photosystem damage linked to an increase in xanthophyll biosynthesis [48,49].

As recently reviewed [50] ALA has an important role in enhancing antioxidant defense. The higher accumulation of ALA by efficient plants enabled a better tolerance to Fe deficiency, probably because ALA is a precursor of heme. Under oxidative stress conditions, heme is released from hemoproteins and forms hemin, its oxidized form [51]. In the present study, hemin accumulation significantly increased in the leaves, particularly in the inefficient plants, being an indicative of higher oxidative stress by these plants. This fact is important since the tetrapyrrole cycle is mainly located in the photosynthetic tissues [10], which is in agreement with the expected higher leaf accumulation levels obtained here. Furthermore, hemin is a form of protoporphyrin IX containing ferric Fe [14] and, when present, it also acts as a strong pro-oxidant in cells because of its participation in H_2_O_2_-dependent redox reactions and to the release of ferric Fe upon its degradation [13,14]. These reactions cause the reduction of molecular oxygen and form ROS [47], thus the observation that inefficient plants had higher oxidative stress. Intracellular accumulation of hemin is highly toxic for cells and plants with low detoxifying ability and cause greater damage, as seems to be the case of inefficient plants. 

When chloroplasts of the mesophyll cells cease to function or are damaged, both anthocyanins and carotenoids have an important photoprotective role, acting as powerful antioxidants [22,52]. Thus, since inefficient plants showed lower levels of these molecules, their capability to manage photooxidation could be hampered. Roots have been shown to accumulate high levels of ROS under mineral stresses [53,54]. MDA levels were higher in the roots of inefficient plants than in efficient plants independently of the Fe conditions, corroborating that the former plants were under higher oxidative damage, since this is an often-used oxidative stress indicator [31,54,55]. This may be due to the very high levels of Fe in the roots of the inefficient plants, which by reaching toxic levels triggered MDA synthesis. On the other hand, there was no significant effect of the Fe availability on MDA accumulation. This result could be perceived as unexpected; however, MDA is one of the final products of lipid peroxidation in the cells and this is usually a reflection of severe oxidative stress [56]. It is possible that our imposed level of Fe deficiency was not severe enough to induce quantifiable changes in lipid peroxidation because of insufficient membrane damage [56].

The membrane-bound FRO enzyme contains the heme-group as a constituent, and it is responsible for the reduction of extracellular Fe, its activity being necessary for Fe uptake [9]. In this study, Fe deficiency did not induce higher levels of FRO activity. Although this is the typical phenotype in Arabidopsis subjected to Fe deficiency [9,57,58], soybean plants oftentimes show a contrasting behavior, as previously shown [8,35,36]. In the current study, this is probably due to the fact that FRO is a heme containing protein and, under Fe deficiency, soybean plants displayed lower levels of heme, as indicated by the lower ALA in the leaves where heme is produced.

FeSOD contains Fe in its active site as a co-factor, indicating a possible interaction with Fe deficiency symptoms. As expected, under Fe deficiency, only the efficient plants were able to maintain the FeSOD activity. This points to a very important role of the FeSOD in the determination of Fe efficiency in soybean. The inability of inefficient plants to maintain FeSOD activity may have led to the observed oxidative stress. This has also been observed in Fe-impaired transgenic tobacco plants which showed decreased FeSOD activity [59]. In Arabidopsis, it has also been demonstrated that Fe deficiency down-regulates FeSOD transcripts [60]. Inefficient plants induced Cu/ZnSOD isoenzymes, which could be a mechanism to replace the lost FeSOD activity. However, current knowledge indicates that FeSOD is indispensable for healthy chloroplast biogenesis and photosynthesis, as shown in Arabidopsis, where plants demonstrated severe albino phenotypes when chloroplastic FeSODs were knocked out [61]. Therefore, loss of FeSOD activity or the inability to maintain control levels by the inefficient plants might have accelerated the chlorosis in this cultivar. 

In the current study, inefficient plants, which had lower Fe accumulation in the leaves and displayed IDC symptoms, had lower APX activity in general. The lower levels of tissue Fe may have impaired APX activity in these plants since APX is a Fe-containing enzyme. APX activity has been shown to be lower in Fe-deficient conditions [62] as a consequence of insufficient Fe availability for the enzyme, as it contains, in addition to the heme group, another Fe atom [62]. This observation has been reported for pea, pear, and quince [62,63]. Together, low APX and FeSOD activity renders inefficient plants unable to deal with the high levels of ROS (increased hemin and MDA in the roots) when compared to the efficient ones. 

Unlike APX, CAT was upregulated under Fe stress in inefficient plants. Previous studies have reported that when there is an upregulation of CAT, a downregulation of APX may occur [64]. Both enzymes are responsible for the conversion of H_2_O_2_ into water, however, while CAT is able to directly reduce H_2_O_2_ into the water with no energy consumption. Despite the fact that lower Fe availability decreases both APX and CAT activity [17,27,30], unlike CAT, APX activity requires ascorbate as a reducing equivalent, being a more energy demanding reaction [30]. This could explain why inefficient plants focus on inducing CAT for oxidative stress defense, presenting lower levels of APX under Fe deficiency. Fe stress increased total POX activity in roots of efficient plants. The regulation of the heme-dependent POX enzymes is influenced by the Fe availability since heme itself requires Fe for proper functioning [21,28]. Inefficient plants were able to maintain relatively high levels of POX activity, because not all peroxidases have a heme group [65]. Still, isoenzymes were differentially regulated in both cultivars. In particular, the accentuated decreases in POX8 and 9 in Fe-deficient leaves of both cultivars deserve further investigation. The substrate used in the current assay (3-3’-diaminobenzidine) is not specific, and does not allow to discriminate neither the physiological role of the enzymes nor their cellular localization.

In the current study, the inefficient plants showed high levels of GR in the leaves, but the concentration of GR in the efficient plants remained at low levels. As the inefficient cultivar accumulated higher ROS levels, triggering the antioxidant system, it seems that GR was particularly induced since it does not contain a heme group in its composition. As such, this enzyme seems to be a good marker for low Fe efficiency in soybean. Glutathione reductase (GR) may allow plants to cope with Fe deficiency in several ways. It is responsible for the reduction of glutathione disulfide to glutathione (GSH), which in turn is able to scavenge H_2_O_2_ through the ascorbate-glutathione cycle [66]. As suggested by others, the up-regulation of the antioxidant system has a direct effect on peroxidative conditions, particularly GR that, as shown here, contributes directly to the decrease in TBARS accumulation and improved defense to Fe deficiency stress [67,68]. Also, GR may aid plants in coping with Fe deficiency through the glutathione peroxidase (GPX) cycle, in combination with SOD [32]. Finally, GR may also play an indirect role in modulating internal Fe homeostasis via nitric oxide (NO) mobilization of Fe [69] or via the dinitrosyl-diglutathionyl–Fe complex [70].

The PCA performed here (Figure 10) shows that the efficient and inefficient lines have distinct behaviors and are clearly separated. Moreover, while in the inefficient plants group there was no separation between +Fe and −Fe treatments, the efficient plants group was clearly divided into two sub-groups correspondent to the Fe treatment showing that inefficient plants are less responsive to Fe stress, and even when Fe is supplied they are unable to utilize it in their metabolism. It is evident that high hemin levels are highly correlated to the inefficient plant trait, which could be key to explain the trait of inefficiency: as these plants are unable to reduce the oxidative stress caused by Fe deficiency, heme molecules are oxidized and, consequently, unavailable to integrate FRO [14] and other Fe metabolism-related enzymes such as SOD and APX. This could explain the lower levels of FRO induction by inefficient plants, observed here and suggested before [36]. The presence of increased hemin levels could have led to more oxidative stress, particularly in the roots, and inefficient plants only seem to trigger the low substrate affinity enzyme CAT. Also, inefficient plants, in an attempt to deal with oxidative stress, were correlated to GR activity at the leaf level. Additionally, Figure 10 displays the previously discussed correlation of the efficiency trait with the antioxidant pigments, leaf MDA accumulation, and APX accumulation.

## 4. Methods

### 4.1. Plant Material and Growth Conditions

One Fe efficient (PI437929 / VIR 316) and one Fe inefficient (PI378676A / Primorskaja 500) *G. max* accession [7], with identical phenology, were selected from the USDA (United States Department of Agriculture) germplasm collection via GRIN (Germplasm Resources Information Network) [71] Seeds were germinated for seven days in the dark, at 25 °C. Germinated seedlings were transferred to 5 L vessels containing hydroponic solution with different Fe treatments. Each vessel contained five plants (*n* = 5) of one accession grown under Fe sufficiency (+Fe, 20 μM Fe (III)-EDDHA [ethylenediamine-*N*,*N*’*bis*(*o*-hydroxyphenyl)acetic acid]) or Fe deficiency (−Fe, no Fe).

The vessels were placed in a climate chamber (Aralab Fitoclima 10000EHF) with 16-h day photoperiod providing 325 μmol s^−1^ m^−2^ of photosynthetic photon flux density at plant level, supplied by a mixture of incandescent bulbs and fluorescent lights. Temperatures were set to 25 °C during the light period and to 20 °C during the dark period, and relative humidity was maintained at 75% throughout the day and night. The standard solution for hydroponic growth of *G. max* included: 1.2 mM KNO_3_; 0.8 mM Ca(NO_3_)_2_; 0.3 mM MgSO_4_.7H_2_O; 0.2 mM NH_4_H_2_PO_4_; 25 μM CaCl_2_; 25 μM H_3_BO_3_; 0.5 μM MnSO_4_; 2 μM ZnSO_4_·H_2_O; 0.5 μM CuSO_4_.H_2_O; 0.5 μM MoO_3_; 0.1 μM NiSO_4_. Hydroponic solution was buffered with 1mM MES [2-(*N*-morpholino)ethanesulfonic acid], pH 5.5 and, during the experimental time, pH was measured and solutions were changed every three days. The experiment ended 14 days after transferring the plants to the climate chamber, when soybean plants usually show the most contrasting symptoms of IDC [72]. Plants were harvested, frozen immediately in liquid nitrogen, and stored at −80 °C for further analysis.

### 4.2. Fe Determination by ICP-OES

A total of 100 mg of dried plant tissue (root and trifoliate leaves) was mixed with 5 mL of 65% HNO_3_ in a Teflon reaction vessel and heated in a SpeedwaveTM MWS-3+ (Berghof, Germany) microwave system. Each plant organ from all the treatments (*n* = 5) was ground and five independent digestions were carried out.

The digestion procedure was conducted in five steps, consisting of different temperature and time sets: 130 °C/10 min, 160 °C/15 min, 170 °C/12 min, 100 °C/7 min, and 100 °C/3 min. The resulting solutions of the digestion procedure were then brought to 20 mL with ultrapure water and filtered for further analysis. Mineral concentration determination was performed using inductively coupled plasma optical emission spectrometer (ICP-OES) Optima 7000 DV (PerkinElmer, USA) with radial configuration.

### 4.3. ALA, Hemin, and Photosynthetic Pigments Evaluation

Protocols for ALA quantification in the leaves were optimized based on protocols previously published [11]. In short, frozen samples (*n* = 5) were ground with liquid nitrogen, using a mortar and pestle, and 200 mg of ground sample was suspended in 1.5 mL of 20 mM potassium phosphate buffer (pH = 6.8). After centrifuging for 10 min at 16,000*g*, 400 µL of the supernatant were mixed with 100 µL of acetylacetone. The mixture was incubated for 10 min at 100 °C and then transferred to room temperature (RT), until cool. At this point, 500 µL of modified Ehrlich’s reagent were added to each sample, the mixture was let to stand for 5 min, and then centrifuged for another 5 min at 16,000*g*. Absorbance was read at 553 nm and ALA concentration was calculated according to a standard calibration curve of 5-aminolevulinic acid hydrochloride (Sigma-Aldrich, #A3785).

Estimated heme protein content in leaves and roots (*n* = 5) was performed by measuring the oxidized version of this protein, hemin, using an enzymatic assay kit (Hemin Assay Kit; Sigma-Aldrich) following the manufacturer instructions.

Chlorophyll, anthocyanin, and carotenoid concentrations were measured on the last fully expanded trifoliate leaf of plants grown in the previously described conditions (*n* = 5). The referred compounds were extracted and quantified according to a modified protocol [73]. Briefly, 0.1 g of leaves ground with liquid nitrogen, were extracted with 10 ml of cold acetone/Tris buffer solution at 1 M (80:20 vol:vol, PH = 7.8). Samples were incubated at 4 °C for 72 h and absorbance values were recorded at 470, 537, 647, and 663 nm. The amount of anthocyanins, chlorophyll *a* and *b*, and carotenoids were determined according to previously published equations [73].

### 4.4. Lipid Peroxidation

To evaluate the lipid peroxidation, thiobarbituric acid reactive substances (TBARS) were measured using a colorimetric adapted method [74]. In short, 0.1 g of roots or trifoliate leaf samples (*n* = 5) were homogenized in 10 mL of 0.5% thiobarbituric acid in 20% trichloroacetic acid (w/v) and incubated at 100 °C for 30 min. The reaction was stopped on ice and samples were centrifuged at 5000 rpm for 10 min. The supernatant was filtered, absorption was read at 450, 532, and 600 nm, and MDA concentration (µmol g^−1^) was calculated from: 6.45 × (A_532_ − A_600_) − 0.56A_450_.

### 4.5. Enzymatic Activity

#### 4.5.1. Root Iron Reductase Activity

Root FRO activity was quantified as previously described by [72]. The measurements were carried out in roots of intact plants (*n* = 5) via the spectrophotometric determination of Fe^2+^ chelated to BPDS (bathophenanthroline disulfonic acid). Roots of each plant were submerged in assay solution containing: 1.5 mM KNO_3_, 1 mM Ca(NO_3_)_2_, 3.75 mM NH_4_H_2_PO_4_, 0.25 mM MgSO_4_, 25 µM CaCl_2_, 25 µM H_3_BO_3_, 2 µM MnSO_4_, 2 µM ZnSO_4_, 0.5 µM CuSO_4_, 0.5 µM H_2_MoO_4_, 0.1 µM NiSO_4_, 100 µM Fe(III)-EDTA (ethylenediaminetetraacetic acid), and 300 µM BPDS. All nutrients were buffered with 1 mM MES, pH = 5.5. The assays were conducted under dim light conditions at 20 °C and were terminated after 45 min by removal of the roots from the assay solution. Absorbance values were obtained spectrophotometrically at 535 nm, and an aliquot of the solution that had no roots during the assay was used as a blank. Rates of reduction were determined using the molar extinction coefficient of 22.14 mM^−1^cm^−1^.

#### 4.5.2. SOD and POX Activity

Enzyme extraction was performed at 4 °C as described in [75]. SOD and POX activity and gel staining of their isoenzymes were carried out according to [76] and references therein. SOD activity was measured by monitoring photochemical reduction of nitroblue tetrazolium (NBT) at 560 nm. The amount of enzyme that inhibited 50% NBT photoreduction was defined as one unit of SOD. POX activity was measured with reaction mixture that contained 3,3-diaminobenzidine, 0.1% (w/v) gelatine in 150 mM Na-phosphate-citrate buffer (pH = 4.4). Reaction was started by the addition of H_2_O_2_ with 0.6% final concentration. Change in absorbance at 465 nm was followed for 1 min. A unit of POX activity was defined as µmol H_2_O_2_ decomposed ml^−1^ min^−1^.

For separation of SOD and POX isoenzymes 12.5% and 10% native separating gels were used respectively. Equal amounts of proteins (50 µg) were loaded to each lane. SOD activity in gels were detected by photochemical staining in the presence of riboflavin and NBT. The different types of SOD were differentiated by incubating gels in inhibitors of SOD prior to NBT staining, such as 2 mM KCN to inhibit Cu/ZnSODs and 3 mM H_2_O_2_ to inhibit Cu/ZnSODs and FeSODs (MnSOD activity is resistant to both). For staining of POX activity, gels were incubated for 30 min in 200 mM Na-acetate buffer (pH = 5.0) containing1.3 mM 3,3-diaminobenzidine and 3% H_2_O_2_ [76]. Gels stained for SOD and POX were photographed with Vilber Lourmat gel documenting system and then analyzed with ImageJ. Isoenzymes in each gel were numbered according to their migration distance. Table S1 from Supplementary Material shows a list of enzymes investigated in this work accompanied with the reactions they catalyze, their EC numbers, and genes in soybean that encode proteins related to these specific EC functions. Soybean genome annotation v1.1 was searched for specific enzyme functions (by using EC no as search string) and genes encoding related proteins were listed. Reactions related to specific EC functions were taken from EXPASY database.

#### 4.5.3. CAT and APX Activity

For the evaluation of CAT and APX activity, an enzymatic extraction was performed according to [77]. Roots and trifoliate leaf samples were analyzed separately (*n* = 5) and 100 mg of ground tissue were homogenized with 1.5 mL of extraction buffer composed of 0.1 M potassium phosphate buffer (pH = 7.0), 0.1 mM EDTA, and 1% polyvinylpyrrolidone. Samples were vortexed for 2 min and then centrifuged for 10 min at 5000 rpm at 4 °C. The supernatant was collected and diluted three-fold. CAT was measured using 666 µL of the diluted supernatant, to which 334 µL of 73 mM H_2_O_2_ in 0.5 M Tris-HCl buffer (pH 7.0) was added. Absorbance was read for 3 min at 240 nm and CAT activity was calculated according to [78]. APX was measured using 100 µL of the initial supernatant, to which 450 µL of 25 mM ascorbic acid and 450 µL of 17 mM H_2_O_2_ in 0.5 M Tris-HCl buffer (pH = 7.0) were added. Absorbance was measured for 3 min at 290 nm and APX activity was calculated according to [79].

#### 4.5.4. GR Activity

For GR, 100 mg of ground roots and trifoliate leaf tissue (*n* = 5) was homogenized with 1.5 mL of extraction buffer containing 50 mM Tris-HCl (pH = 7.5) and 1 mM EDTA. The mixture was vortexed for 2 min and centrifuged for 10 min at 5000 rpm at 4 °C. To 100 µL of the supernatant, 1 mL of a solution containing 1 mM EDTA, 0.5 mM GSSSG, 0.15 mM NADPH, 50 mM Tris-HCl buffer (pH = 7.5), and 3 mM MgCl_2_ was added to each sample. Absorbances were read for 1 min at 340 nm and GR activity was calculated according to [80].

#### 4.5.5. Statistical Analysis

Data were analyzed with GraphPad Prism version 6.00 for Mac OS X (GraphPad Software, La Jolla California USA, www.graphpad.com). Differences among all groups (treatments and cultivars) were tested with two-way ANOVA corrected for multiple comparisons using Tukey method. Statistical significance was considered at *p* < 0.05.

Principal component analysis (PCA) was performed to establish the relationships among the different variables. The data set included 16 continuous variables, namely, the concentration of anthocyanins, total chlorophylls, carotenoids, leaf ∂-aminolevulinic acid, root ∂-aminolevulinic acid, leaf hemin, root hemin, leaf MDA and root MDA; and the activity of leaf APX, root APX, leaf GR, root GR, root FRO, leaf CAT and root CAT. This analysis was performed using Tanagra data mining software, version 1.4.5 (Lyon, France) [81].

## 5. Conclusions

Efficient plants under Fe sufficiency do not over accumulate Fe in the roots, and are able to transport adequate levels of Fe to the shoots, thus avoiding IDC. They adjust their pigment concentration under Fe stress to lower photoinhibition and consequently ROS levels while inefficient plants are not able to do so. As such, lower stress levels are sensed by the upper organs and MDA concentrations remain unaltered in root and shoot tissues, independent of Fe supply. ALA remains available in the leaves for chlorophyll synthesis and the heme-group continues to be available for enzyme integration. In this way, FRO activity is higher in efficient plants when compared with the inefficient ones and FeSOD activity is maintained, even under Fe deficiency. Efficient plants do not need to activate their antioxidant system because there is no formation of reactive oxygen species under iron deficiency.

**Figure 11:**
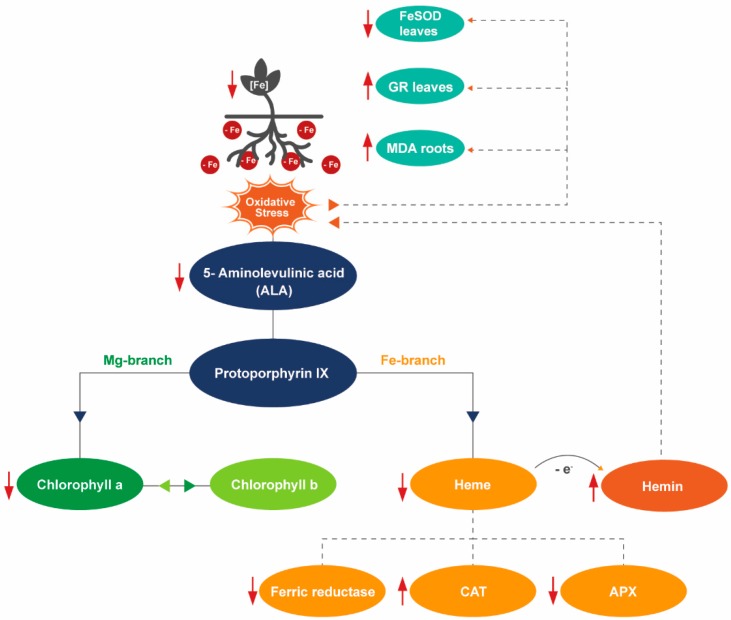
Proposed scheme for antioxidant responses and tetrapyrrole metabolism regulation of inefficient (INF) plants under Fe deficiency (−Fe). Full lines connect the main components of the tetrapyrrole cycle; dashed lines represent the influence of one product on another; red arrows represent increased (up) or decreased (down) concentration of a certain product.

In Figure 11 we summarize the antioxidant responses and tetrapyrrole metabolism regulation associated to the inefficient plants when exposed to Fe deficiency. This study shows that inefficient plants are unable to transport adequate levels of Fe to the shoots, resulting in IDC symptoms. They have lower photosynthetic pigments concentration when compared to the efficient plants and are unable to adjust to the photosynthetic pigment concentration under Fe stress, leading to photoinhibition. High levels of ROS are then accumulated and high oxidative stress levels are imposed to the plant, leading to MDA and hemin accumulation. ALA levels are reduced, leading to lower production of heme, which in turn impairs APX, FeSOD, and FRO activity. At the root level, CAT is induced, as this is the less energy requiring antioxidant enzyme and, at the leaf level, GR activity is induced, as this is a non-heme and non-Fe containing enzyme.

## Supplementary Materials

The following are available online at https://www.mdpi.com/2223-7747/8/9/348/s1, Figure S1: List of enzymes investigated in this work accompanied with reactions they catalyze, their EC numbers, and genes in soybean that encode proteins related to these specific EC functions. Soybean genome annotation v1.1 was searched for specific enzyme functions (by using EC no as search string) and genes encoding related proteins were listed. Reactions related to specific EC functions were taken from EXPASY database.
