# Supplementary material for: Understanding the Role of the Antioxidant System and the Tetrapyrrole Cycle in Iron Deficiency Chlorosis
Source: Plants (Basel). 2019 Sep 13;8(9):348. doi: 10.3390/plants8090348 (PMC6784024; doi:10.3390/plants8090348)
Supplement: Supplementary file 1 [file plants-08-00348-s001.pdf]

Table S1. List of enzymes investigated in this work accompanied with reactions they catalyse, their EC numbers and genes in soybean that encode proteins related to these specific EC functions. Soybean genome annotation v1.1 was searched for specific enzyme functions (by using EC no as search string) and genes encoding related proteins were listed. Reactions related to specific EC functions were taken from EXPASY database.

**Enzyme/Reaction catalysed**	**E.C. no**	**Genes related to function**
Superoxide dismutase (SOD)<br>2 superoxide + $2H^+$ <=> O_2 + H_2O_2	1.15.1.1	Glyma03g40280,<br>Glyma04g39930,<br>Glyma06g14960,<br>Glyma11g19840,<br>Glyma12g08650,<br>Glyma12g30260,<br>Glyma16g27020,<br>Glyma19g42890
Catalase (CAT)<br>2 H_2O_2 <=> O_2+ 2 H_2O	1.11.1.6	Glyma04g01920,<br>Glyma06g02040,<br>Glyma14g39810,<br>Glyma17g38140
Peroxidase (POX)<br>2 phenolic donor + H_2O_2 <=> 2 phenoxyl radical of the donor + 2 H_2O	1.11.1.7	Glyma01g09650,<br>Glyma01g37630,<br>Glyma01g39080,<br>Glyma01g39990,<br>Glyma02g14090,<br>Glyma02g15280,<br>Glyma02g15290,<br>Glyma03g01010,<br>Glyma03g01020,<br>Glyma03g30180,<br>Glyma04g39860,<br>Glyma05g22180,<br>Glyma06g06350,<br>Glyma06g15030,<br>Glyma06g28890,<br>Glyma07g33180,<br>Glyma07g36580,<br>Glyma07g39020,<br>Glyma07g39290,<br>Glyma08g09310,<br>Glyma08g19180,<br>Glyma08g40280,<br>Glyma09g00480,<br>Glyma09g02600,<br>Glyma09g02610,<br>Glyma09g06350,<br>Glyma09g07550,<br>Glyma09g16810,<br>Glyma09g27390,<br>Glyma09g28460,<br>Glyma10g05800,<br>Glyma10g36680,<br>Glyma10g38520,

		Glyma11g05300,<br>Glyma11g06180,<br>Glyma11g07670,<br>Glyma11g10750,<br>Glyma11g30010,<br>Glyma12g32160,<br>Glyma12g32170,<br>Glyma12g37060,<br>Glyma13g00790,<br>Glyma13g04590,<br>Glyma13g16590,<br>Glyma13g20170,<br>Glyma13g23620,<br>Glyma13g24110,<br>Glyma13g38300,<br>Glyma13g38310,<br>Glyma13g42140,<br>Glyma14g07730,<br>Glyma14g12170,<br>Glyma14g40150,<br>Glyma15g03250,<br>Glyma15g05810,<br>Glyma15g05820,<br>Glyma15g13500,<br>Glyma15g13510,<br>Glyma15g13550,<br>Glyma15g16710,<br>Glyma15g17620,<br>Glyma16g06030,<br>Glyma16g33250,<br>Glyma17g01440,<br>Glyma17g01720,<br>Glyma17g04030,<br>Glyma17g06080,<br>Glyma17g06090,<br>Glyma17g06890,<br>Glyma17g33730,<br>Glyma17g37240,<br>Glyma18g06210,<br>Glyma19g01620,<br>Glyma20g30910,<br>Glyma20g31190,<br>Glyma20g33340
Ascorbate peroxidase (APX)<br>2 L-ascorbate + H_2O_2+ 2 H^+ <=> L-ascorbate + L-dehydroascorbate + 2 H_2O	1.11.1.11	Glyma02g37160,<br>Glyma04g42720,<br>Glyma06g07180,<br>Glyma06g12020,<br>Glyma11g11460,<br>Glyma11g15680,<br>Glyma12g03610
Glutathione reductase (GR)	1.8.1.7	Glyma02g08180,<br>Glyma02g16010,

glutathione disulfide + NADPH <=> 2 glutathione + $NADP^+$		Glyma10g03740, Glyma16g27210
